# Synthetic Derivatives against Wild-Type and Non-Wild-Type *Sporothrix brasiliensis*: In Vitro and In Silico Analyses

**DOI:** 10.3390/ph15010055

**Published:** 2022-01-01

**Authors:** Lais Cavalcanti dos Santos Velasco de Souza, Lucas Martins Alcântara, Pãmella Antunes de Macêdo-Sales, Nathália Faria Reis, Débora Sena de Oliveira, Ricardo Luiz Dantas Machado, Reinaldo Barros Geraldo, André Luis Souza dos Santos, Vítor Francisco Ferreira, Daniel Tadeu Gomes Gonzaga, Fernando de Carvalho da Silva, Helena Carla Castro, Andréa Regina de Souza Baptista

**Affiliations:** 1 Center for Microorganisms’ Investigation, Fluminense Federal University, Niterói 24020-150, Brazil; laiscavalcanti@id.uff.br (L.C.d.S.V.d.S.); martins_lucas@id.uff.br (L.M.A.); pantunes@id.uff.br (P.A.d.M.-S.); nathaliafariareis@id.uff.br (N.F.R.); dsena@id.uff.br (D.S.d.O.); ricardomachado@id.uff.br (R.L.D.M.); 2 Laboratory of Antibiotics, Biochemistry and Molecular Modeling, Institute of Biology, Fluminense Federal University, Niterói 24210-201, Brazil; reinaldobgeraldo@gmail.com (R.B.G.); hcastrorangel@yahoo.com.br (H.C.C.); 3 Laboratory of Advanced Studies of Emerging and Resistant Microorganisms, Federal University of Rio de Janeiro, Rio de Janeiro 21941-902, Brazil; andre@micro.ufrj.br; 4 Department of Pharmaceutical Technology, Faculty of Pharmacy, Graduate Program in Applied Health Sciences, Niterói 24241-000, Brazil; vitorferreira@id.uff.br; 5 Department of Pharmaceutical Technology, Faculty of Pharmacy, Fluminense Federal University, Niterói 24241-000, Brazil; 6 Pharmacy Unit, State University of the West Zone, Rio de Janeiro 23070-200, Brazil; danieltadeugonzaga@yahoo.com.br; 7 Department of Organic Chemistry, Institute of Chemistry, Fluminense Federal University, Niterói 24020-150, Brazil; fcsilva@id.uff.br

**Keywords:** sporotrichosis, *Felis catus*, quinones, hydrazones, zoonoses

## Abstract

Recently, the well-known geographically wide distribution of sporotrichosis in Brazil, combined with the difficulties of effective domestic feline treatment, has emphasized the pressing need for new therapeutic alternatives. This work considers a range of synthetic derivatives as potential antifungals against *Sporothrix brasiliensis* isolated from cats from the hyperendemic Brazilian region. Six *S. brasiliensis* isolates from the sporotrichotic lesions of itraconazole responsive or non-responsive domestic cats were studied. The minimum inhibitory concentrations (MICs) of three novel hydrazone derivatives and eleven novel quinone derivatives were determined using the broth microdilution method (M38-A2). In silico tests were also used to predict the pharmacological profile and toxicity parameters of these synthetic derivatives. MICs and MFCs ranged from 1 to >128 µg/mL. The ADMET computational analysis failed to detect toxicity while a good pharmacological predictive profile, with parameters similar to itraconazole, was obtained. Three hydrazone derivatives were particularly promising candidates as antifungal agents against itraconazole-resistant *S. brasiliensis* from the Brazilian hyperendemic region. Since sporotrichosis is a neglected zoonosis currently spreading in Latin America, particularly in Brazil, the present data can contribute to its future control by alternative antifungal drug design against *S. brasiliensis*, the most virulent and prevalent species of the hyperendemic context.

## 1. Introduction

Sporotrichosis is a post-traumatic implantation subcutaneous mycosis of worldwide occurrence and increasing incidence, especially in tropical and subtropical regions such as Asia and Latin America. In Brazil, it is a hyperendemic neglected zoonosis and a major public health concern [1,2]. The pathogenic fungi causing this dermatozoonosis, from the genus *Sporothrix*, are thermo-dimorphic and saprophytic microorganisms [2]. *Sporothrix brasiliensis* is the most prevalent species in Brazil, directly related to the cat-transmitted zoonotic route [2,3]. The recent detection of feline and human patients with *S. brasiliensis*-caused sporotrichosis in Argentina [4,5,6] in addition to reports of zoonotic cases in other countries such as Paraguay and Panamá [7,8]. Suspected or possible cases were detected in Bolivia and Colombia, revealing its potential dissemination across South America [9]. One of the main challenges to be addressed is the occurrence of severe non-responsive forms of this mycosis in cats, in addition to recurrences and reinfections, characterizing zoonotic sporotrichosis as a matter of large relevance to public health [3,10].

Currently, the drug of choice for human and animal sporotrichosis treatment is itraconazole [11,12]. This azole is hepatotoxic, exclusively administered orally, with a high cost for the affected population, most of which live in socially vulnerable areas [13]. Thus, despite being improved, sporotrichosis therapy remains an unsolved problem since the reduction in *S. brasiliensis* sensitivity to this azole has been reported [12,14,15]. These authors described *S. brasiliensis* isolates obtained from patients residing in distinct Brazilian states such as Rio de Janeiro [16,17,18], São Paulo [16], Minas Gerais [18] and Rio Grande do Sul [12,14,19]. Our research group recently described the unprecedented existence of domestic felines coinfected by wild-type and non-wild-type *S. brasiliensis* in distinct areas of the hyperendemic region [17]. The ability to adapt and manifest the phenomenon of antifungal resistance to conventional antifungals is a reality for *Sporothrix* species originating from human and animal cases. Furthermore, *S. brasiliensis* presents a high ability to display resistance mechanisms, although these have not yet been fully elucidated [15].

The pharmaceutical industry is currently seeking new approaches for developing antimicrobial drugs to mitigate the worldwide burden of fungal diseases. In addition, the discovery and commercialization of new antifungal agents occurs in the long term and is costly. With regard to *Sporothrix* spp. infections, a few synthetic derivatives [20,21,22] have been investigated and described in the literature. Additionally, the potential correlation between feline therapeutic follow up with the in vitro itraconazole performance against *S. brasiliensis* has rarely been investigated. The present study aimed to determine the potential antifungal properties of a range of synthetic derivatives against wild-type and non-wild-type *S. brasiliensis* from diseased cats of the Brazilian hyperendemic area.

## 2. Results

### 2.1. Clinical Epidemiological Data

Table 1 summarizes the clinical epidemiological data of the six diseased felines from which the *S. brasiliensis* isolates were obtained. More than half were male (66.7%) and half of the total were unneutered. The average age of these animals was 3.58 years (SD ± 3.47 years), ranging from one to ten years. The majority were free roaming (83.3%), residing in urban areas with contact with soil and/or plants. Lesions were distributed in different anatomical sites, regardless of disease severity and the extent of itraconazole treatment.

Two domestic felines, the carriers of the WT3 and of the NWT1 isolates, presented more than one episode of sporotrichosis relapse. The NWT1 carrier underwent tail amputation 4 years ago and suffered from a new episode of sporotrichosis in the tail stump at the time of this study. Itraconazole treatment ranged from 2 to more than 120 months, with a dosage of 100 mg/cat/day.

### 2.2. Antifungal Susceptibility Assay

**Figure 1:**
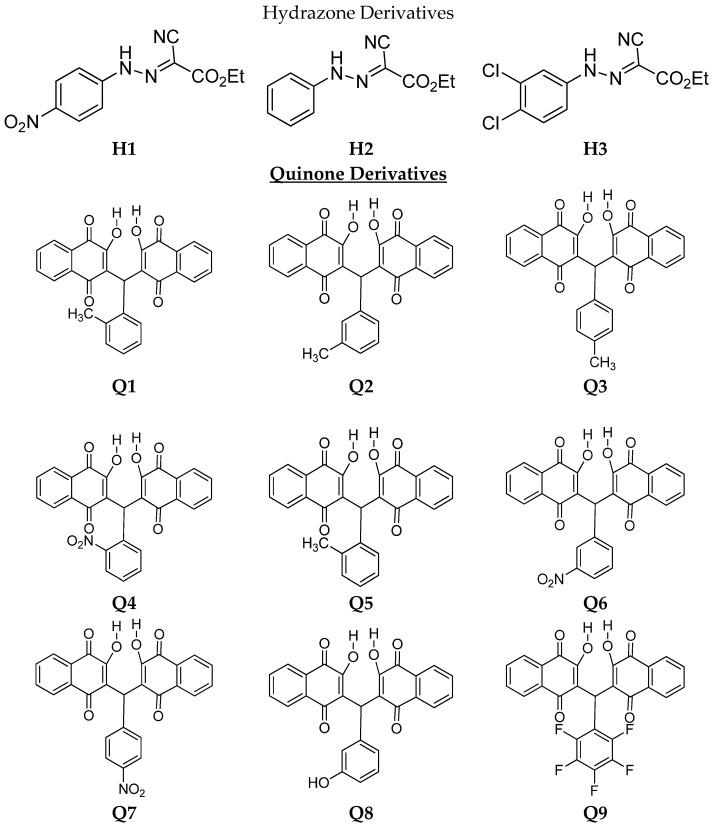

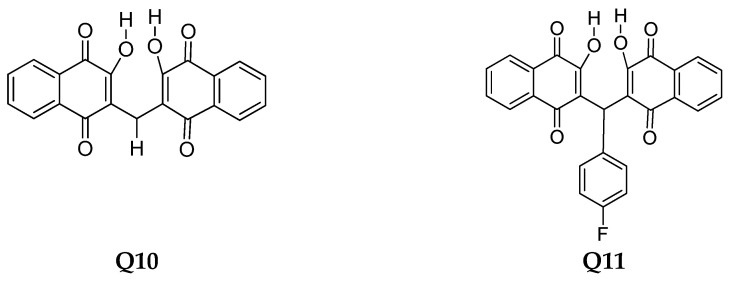
Molecular structures of hydrazone and quinone derivatives.

After the positive initial screening by an adaptation of the diffusion disc method (CLSI M2-A8) [24], eleven quinone derivatives (Q1–Q11) and three hydrazones (H1–H3) were selected for further investigation by the microdilution broth test against *S. brasiliensis* yeast-like forms (Figure 1). The results of the MIC and MFC values of the six clinical isolates and the standard strain against both hydrazone and quinone derivatives as well as itraconazole are shown in Table 2, Table 3 (geometric means to hydrazones) and Table 4.

An itraconazole MIC variation between 1 and 32 µg/mL and an MFC variation between 8 and >128 µg/mL against *S. brasiliensis* were observed (Table 2). Isolates obtained from the lesions of long-term treated cats with recurrent episodes presented the highest MICs for itraconazole (NWT1 MIC = 32 µg/mL; NWT2 MIC = 16 µg/mL and NWT3 MIC = 8 µg/mL) as well as the highest MFCs (Table 2).

*S. brasiliensis* exposure to hydrazones provided MICs varying from 1 to 16 µg/mL (Table 2), while for quinones, the MICs ranged from 32 to >128 µg/mL (Table 4). MICs’ and MFCs’ geometric means (GMs) for the hydrazone derivatives ranged from 2.7 to 13.3 µg/mL (Table 3). The GMs of the MICs obtained for NWT isolates exposed to the three hydrazones were between a third and a half lower (5.7–10.7 µg/mL) than those obtained for itraconazole (Table 3). In contrast, for the quinone derivatives, higher MICs and MFCs were detected (32–>128 µg/mL; Table 4). The clinical isolate NWT1, obtained from a two-year-old domestic feline irresponsive to itraconazole, showing several episodes of relapse, presented in vitro results suggestive of sensitivity to the Q8 (MIC/MFC = 32 µg/mL; Table 4) as well as to H1, H2 and H3 (MIC = 1–8µg/mL; MFC = 1–16 µg/mL; Table 2).

The WT1 clinical isolate from the feline treated with itraconazole for 60 months showed MICs compatible with sensitivity after exposure to itraconazole (1 µg/mL), Q1 (32 µg/mL), H1 (2 µg/mL), H2 (16 µg/mL) and H3 (2 µg/mL). Meanwhile, the MFCs were, respectively, Q1 (32 µg/mL), H1 (2 µg/mL), H2 (16 µg/mL) and H3 (4 µg/mL). WT2 and WT3 exposed to hydrazones exhibited the same suggestive phenotype (Table 2). MICs and MFCs of the quinones were also high for the wild-type isolates (Table 4). According to the MFCs, all hydrazones and quinones presented a fungicidal profile. 

### 2.3. The In Silico Toxicological Profile and Pharmacokinetics

Toxicity was predicted as described in Table 5. The oral rat acute toxicity (LD_50_) of all derivatives and itraconazole ranged from 1.952 to 2.984 mol/kg. In contrast, oral rat chronic toxicity (LOAEL) varied from 0.055 log mg/kg bw/day to 2.974 mg/kg bw/day. Minnow toxicity varied between −6.407 log LC50 and 0.919 log LC50. Q6, Q7, H1 and H3 did not show evidence of a hepatotoxic effect. No hydrazone was a potential inhibitor of HERG I and II channels, while quinones did not seem to inhibit HERG I. The in silico predictions of four toxicological endpoints revealed that Q4, Q11 and itraconazole were immunotoxic, and that Q4, Q5 and H1 were mutagenic. No compound presented any presumed carcinogenic or cytotoxic properties.

The pharmacokinetics profile of THE synthetic derivatives in silico shown in Table S1 summarizes the in silico intestinal absorption analysis of all compounds showing values above 80% (84.81–100%), and most of the derivatives (8/14) exhibited good Caco-2 permeability, similarly to itraconazole. In contrast, Q1, Q7, H2 and H3 were not determined to be P-glycoprotein substrates. No hydrazone derivative was suggested as an inhibitor of P-glycoprotein I/II while Q9 was not assigned as a P-glycoprotein II inhibitor.

The volume of distribution (VDss) showed lower values (−0.807 and −0.169 log L/kg) for almost all derivatives. Likewise, low values were shown for the majority of derivatives with regard to the unbound drug fraction (UF). However, H1, H2, H3 and Q8 compounds presented estimated values ranging from 0.274 to 0.386, above those of itraconazole (Table S1). In addition, H3 was the only derivative with a suggested capability of blood–brain barrier permeability (BBB).

These compounds were also analyzed for interaction with CYP 450 enzymes, as substrates (2D6 and 3A4) or as inhibitors (CYP1A2, CYP2C19, CYP2C9, CYP2D6, CYP3A4). The hydrazone derivatives were not substrates for CYP3A4, and none of them were substrates for CYP2D6. As inhibitors, only the H3 compound showed an inhibitory profile and only against CYP1A2. Q1, Q2, Q3, Q4 and Q10 compounds inhibited CYP2C19, CYP2C9, CYP2D6 and CYP3A4. The candidates showed total clearance activity ranging from −0.926 log mL/min/kg to 0.786 log mL/min/kg. Similarly, no renal OCT2 substrate activity was found for either classes.

## 3. Discussion

The present work investigated *S. brasiliensis* isolates obtained from the lesions of domestic cats with sporotrichosis, clinically classified as responsive or non-responsive to treatment with itraconazole. This treatment varied from 2 to 60 months, and as expected, *S. brasiliensis* showed MICs compatible with reduced sensitivity to itraconazole or the “non-wild-type” phenotype, whenever obtained from non-responsive cats, as previously proposed (ESPINEL-INGROFF et al. [23] Almeida-Paes et al. [25]. The cats’ mean age of approximately 3 year was in accordance with previous reports on sporotrichosis, as was the predominance of males. Indeed, with this species, sexual maturity increases fighting for females and concomitant exposure to other diseased animals [1,2,3].

Although itraconazole is the first therapeutic option for human and domestic feline sporotrichosis treatment [12,14,23], broth microdilution cut-off points have not been established. As a consequence, the definition of qualitative parameters of *Sporothrix* spp. response (sensitivity or resistance) to the different antifungal drugs remains a riddle to be solved. In the attempt to clarify this definition, epidemiological cutoff values (ECVs) were suggested [23,25] though not yet fully correlated to the patients’ clinical responses [12,14,23]. Recently, Nakasu et al. [12] published a single study in an attempt to draw a parallel between in vitro data and the feline therapeutic follow up.

Recent publications have suggested that ECVs with MICs in the range of 0.5–2 mg/L for itraconazole were considered indicative of *S. brasiliensis* in vitro sensitivity [12,14,23,25]. The present study data on *S. brasiliensis* clinical isolates support this indication since those originating from domestic felines with an effective therapeutic response to the azole presented an in vitro MIC of 2 µg/mL. In contrast, the *S. brasiliensis* obtained from animals showing poor therapeutic response to itraconazole provided MIC values between 8 and 32 µg/mL, and could therefore be considered as “non-wild type” [23,25] or even resistant [12,14,15]. Likewise, in a veterinary hospital in southern Brazil (Pelotas, RS), Nakasu et al. [12] showed that approximately half of the investigated cats were non-responsive to itraconazole, with the corresponding isolates “resistant” to this drug. However, the reported MICs for itraconazole detected in the present investigation were higher than those reported by these authors. This might reflect the fact that the South Region of Brazil is located 1800 km away from the hyperendemic area (Rio de Janeiro, Southeast Brazil) with a lower level of sporotrichosis endemicity [1,3].

**Figure 2:**
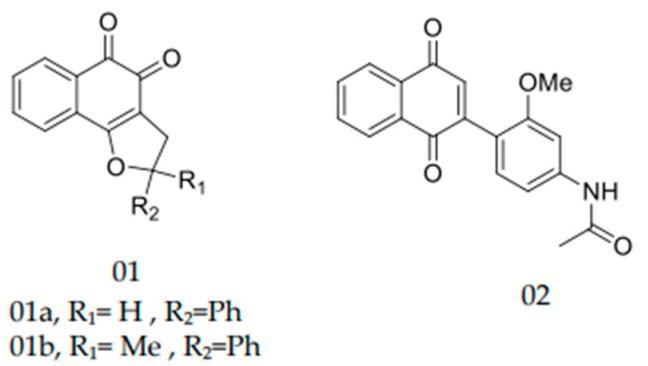
Naphthoquinones which previously presented antifungal activity against *Sporothrix* spp. as published by Janeczko et al. [31].

Different authors have suggested that *S. brasiliensis* shows reduced sensitivity to azoles [12,14,23,25], underlining the need to distinguish therapeutic alternatives. Recently, quinones became the subject of several studies due to their diverse known biological activities, such as their antibacterial [26,27] and antifungal potential [21,28,29]. These drugs also have antifungal properties against *S. schenckii* [29]. The present study analyzed eleven new quinone derivatives against WT and NWT clinical isolates and the standard strain of *S. brasiliensis*. Quinone derivatives Q1 and Q8 exhibited MIC and MFC values compatible with potential antifungal molecules [28,29]. Indeed, Tandon et al. [29,30], assessing quinone derivatives’ antibacterial and antifungal potential, showed MICs between 6.25 and ≥50 µg/mL for distinct *S. schenckii* isolates. Another example of an antifungal naphthoquinone is the substituted α-and-β-2,3-dihydrofuranaphthoquinones which were synthesized and evaluated against the main zoonotic *Sporothrix* species [21]. Two compounds were strongly active against these dimorphic fungi, namely naphthoquinone 1a, (Figure 2) which presented an MIC of 4 mg/mL, and naphthoquinone 1b (Figure 2), which presented an MIC of 2 mg/mL and 4 mg/mL against *S. brasiliensis* and *S. schenckii*, respectively. In the present study, the MICs obtained after the in vitro exposure of *S. brasiliensis* versus quinones were higher than those reported by Ferreira et al. [21], but still compatible with therapeutic potential.

The antifungal activity of naphthoquinones can also be found against other fungi, such as *Candida albicans*. In the work of Janeczko et al. [31], a series of 1,4-naphthoquinones was prepared and tested against *C. albicans*. The compound (2) was the most active with an MIC of 8 mg/mL (Figure 2). The precedents for antifungal activities indicate that this class of compounds has good prospects, especially for hybrids with heterocyclic nuclei. Altogether, these data corroborate the potential of this class of molecules as a source of antifungal drug candidates.

Three hydrazones investigated in the present study also showed potential antifungal activity against different clinical isolates of *S. brasiliensis*. The in vitro exposure to H1 resulted in an MIC of 1 µg/mL against the *S. brasiliensis* NWT1, while the corresponding itraconazole value was 32-fold higher. Remarkably, this isolate infected a cat with a history of prolonged itraconazole-non-responsive sporotrichosis manifested by lesion recurrence at the base of its amputated tail. The standard strain of *S. brasiliensis* (*Sbra*) revealed the same in vitro response to these molecules.

The therapeutic potential for the hydrazones was previously investigated with several described biological activities [32]. Its antimycotic potential against dimorphic fungi, such as *Coccidioides posadasii,* as well as highly relevant yeasts including *Candida* spp. and *Trichosporon asahii*, in both community and hospital-acquired infections [32,33,34,35], were reported. Previously, Cordeiro et al. [34] described hydrazone MICs ranging from 2 to 250 µg/mL against *Coccidioides posadasii*, higher than those reported for *S. brasiliensis* in the present study, as compatible with the further investment of hydrazones as candidate drugs against this dimorphic fungus. Furthermore, distinct *Candida* species challenged this in vitro growth in the presence of different hydrazone derivatives resulting in MICs ranging from 0.25 to 128 µg/mL [35]. Previously, Casanova et al. [33] proposed that an MIC value between 8 and 32 µg/mL could be considered an encouraging result after the in vitro testing of new hydrazones against *Candida parapsilosis* and *Trichosporon asahii*. Our results indicate the strong potential of future investment in hydrazones for controlling fungal infections.

**Figure 3:**
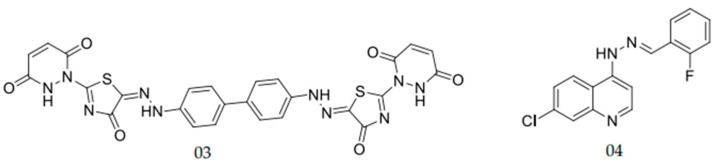
Aryl hydrazones which previously presented antifungal activity against *Candida albicans*, as published by Carvalho et al. and Abu-Melha et al. [36,37].

Hydrazones are present in different aryl groups which confer antifungal activity [36,37]. Abu-Melha et al. [37] performed tests with a one bis aryl hydrazine containing a thiazole ring (Hydrazone 03; Figure 3) against *C. albicans*. This compound resulted in MICs of 0.18 µg/mL and 3.18 µg/mL against azole-sensitive and azole-resistant *C. albicans*, respectively [37]. Carvalho et al. [36] evaluated a series of hydrazones containing the nucleus 7-chloroquinoline. The hydrazone shown in Figure 3 (hydrazone 04) stood out with a high percentage of inhibition against *C. albicans* (71% growth inhibition at a concentration of 12.5 mg/mL).

Hydrazone and quinone derivatives were submitted to in silico ADME/Tox property analyses. Absorption is the process whereby the drug candidate moves from the point of administration (extravascular site) to the blood (systemic circulation). The values above 90%, present an optimal intestinal profile [38]. In the present work, all tested derivatives revealed good theoretical absorption in the intestine, similarly to itraconazole. Moreover, some quinone derivatives and itraconazole presented permeability to Caco-2 cells [39]. The Caco-2 in silico assay is a useful feature to analyze the passive absorption in the intestine, so the hydrophobic parameters shows the most relevance in this relationship [40]. Thus, parameters such as hydrophobic (LogP), molecular polar surface area (TPSA), molecular weight and donor/acceptor of hydrogen may influence Caco-2 results. Therefore, due to the hydrophilic profile of the quinolone derivatives such as Q4, Q5 and Q11 and of the hydrazone derivatives (H1–H3), these did not show permeability profiles to the in silico Caco-2 assay.

One last absorption parameter was analyzed by employing the P-glycoprotein transporters’ substrate or inhibitor [41]. The non-linear absorption kinetics of P-glycoprotein substrates has been reported due to the saturation transporter-mediated efflux activity, promoting the inhibition of intestinal P-glycoprotein resulting in significant effects of drug interaction (DDI) [42,43]. The results suggested that Q1, Q7, H2 and H3 derivative compounds were not P-glycoprotein substrates suggesting a good in silico oral bioavailability for all analyzed derivatives to be further explored.

The volume of distribution (VDss), fraction unbound (human) and blood–brain barrier membrane (BBB) permeability were also analyzed, as a distribution parameter for all the studied compounds. The VDss indicates the theoretical volume that a total dose would need to be uniformly distributed in the plasma to obtain the same concentration observed in blood plasma. All compounds showed lower values of VDss, suggesting that all these prototypes need low concentrations to remain in blood plasma. A fraction of the unbound drug of all hydrazones is higher than that of itraconazole. These results indicate that these groups of molecules have a good predictive distribution because the unbound form of the drug is responsible for exercising pharmacological activity [44]. The blood–brain barrier (BBB) was analyzed and according to the SwissADME calculation, only the H3 derivative could pass through the blood–brain barrier.

Enzymatic metabolism indicates the chemical biotransformation of a drug in the body, which plays a vital role in converting drug compounds [43]. CYP enzymes represent the most studied phase I drug-metabolizing enzymes and are also implicated in drug–drug interactions (DDIs) mediated by drug inhibition [45]. Although none of the derivatives were substrates for the tested Cytochrome P450 isozyme 2D6, all quinones are substrates of 3A4, such as itraconazole. Additionally, among these derivatives, only H3 acts as an inhibitor for CYP1A2. Interestingly, CYP1A2 is one main xenobiotic-metabolizing enzyme in humans, and a recent study associated this enzyme with the bioactivation of procarcinogens, including 4-(methylnitrosamino)-1-(3-pyridyl)-1-butanone (NNK), a tobacco-specific and potent pulmonary carcinogen [43,45].

Clearance is a constant that describes the relationship between drug concentration in the body and is important in determining the elimination of the drug [46]. Apart from hydrazone derivatives, all other new compounds exhibited lower values, including itraconazole. These metabolic and excretion results indicated that, theoretically, the quinone derivatives present a hepatic metabolization, while the hydrazone derivatives present a renal metabolization.

Toxicity has been a significant concern to the safety of drug candidates. Hepatotoxicity is still one of the major problems of drug toxicity [47]. Consequently, only Q6, Q7, H1 and H3 derivatives showed no predicted hepatotoxicity. Concerning the in silico toxicity test with minnows, an equivalent lethal concentration value (LC_50_), representing the concentration of a molecule necessary to cause the death of 50% of experimentally tested Fathead minnows, LC_50_ < 0.5 mM (i.e., log LC_50_ < −0.3) is regarded as causing acute toxicity. Q6, Q7, H1 and H2 derivatives do not seem to present acute toxicity in the minnow test. All quinones—apart from Q8—and hydrazones proved to be less toxic than itraconazole. No hydrazone was potentially an inhibitor of HERG I and II channels [48], while quinones did not seem to inhibit HERG I, avoiding fatal heart-beat problems and short QT syndrome.

The oral rat acute toxicity expresses the compound toxic potential in terms of lethal dosage values (LD_50_ in mol/kg). These chronic toxicity studies aim to detect the lowest doses of a compound that will cause the lowest observed adverse effect levels (LOAELs), and the treatment period and exposure time of the compound must also be considered [49]. Log LOAEL predicted values for the tested compounds suggest that a larger dose of each compound must be used to induce adverse effects, indicating safety for these compounds compared to itraconazole.

ProTox-II proposes to classify drugs into several different steps, including a toxicological (immunotoxicity model), genotoxicological (cytotoxicity, mutagenicity and carcinogenicity model) endpoints, and the toxicity of a specific protein target [50,51]. The in silico results revealed that all compounds do not present cytotoxicity and carcinogenicity. In general, theoretical toxicity studies show that the derivatives were less toxic than the antifungal of choice.

## 4. Materials and Methods

### 4.1. Fungal Isolates

Six clinical *S. brasiliensis* isolates were obtained from the lesions of cats with laboratory-confirmed sporotrichosis. The clinical evaluations were performed by veterinarians, and the six cats’ lesions, suspected to be from sporotrichosis, were later confirmed by mycological culture isolation and the genotyping of *S. brasiliensis*; all cats resided in the hyperendemic region of Rio de Janeiro, Brazil, as previously described [3]. These were selected from the Center for Microorganisms’ Investigation fungal collection, based on the itraconazole in vitro results [52,53,54] following the criteria proposed by Espinel-Ingroff et al. [23], as wild type (MIC ≤ 2µg/mL) or non-wild type (MIC ≥ 4µg/mL). These were further designated as “WT1”, “WT2”, “WT3” and “NWT1”, “NWT2”, “NWT3”, respectively. The reference strain *S. brasiliensis* ATCC MYA-4823 (*Sbra*) was included in all experiments.

### 4.2. Growth Conditions

The clinical isolates were maintained by cryopreservation in its yeast phase at −20 °C in the fungus collection of the Center for Microorganism’s Investigation (CIM) until its reactivation for the conduction of the experiments. For reactivation, cryotubes were defrosted with subsequent replication on Sabouraud agar 2% dextrose (Becton, Dickinson, and Company—BD, Franklin Lakes, NJ, USA) and incubated at room temperature for five days for microbial growth in the form of conidia.

### 4.3. Synthetic Derivatives

Seventy-eight novel chemical derivatives of seven different classes (pyrazoles, pyrazolones, quinolones, naphthoquinones, hydrazones, n-phthalimides and quinones) were screened by disk diffusion antimicrobial sensitivity test (adapted Kirby–Bauer methodology) [55].

### 4.4. Antifungal Susceptibility Assays

Susceptibility testing was performed according to the standardized broth microdilution technique described by the CLSI in documents M38-A2 and M27-A3 [52,53] for yeast-like cells and conidia. The antifungal used as the experimental control was itraconazole.

Minimum fungicidal concentrations (MFCs) were obtained from subcultures on Petri dishes, including Sabouraud agar 2% dextrose (SDA; Becton, Dickinson and Company—BD, Franklin Lakes, NJ, USA), filamentous phase and brain heart infusion (BHI; Becton, Dickinson and Company—BD, Franklin Lakes, NJ, USA), yeast phase, with 30 µL of the MIC cell suspension. In plates, they were incubated at 25 °C for five days (conidia) and at 37 °C for seven days (yeast). After reading the number of colonies, the MFC was established as the lowest derivative concentration capable of eliminating 99.9% of the fungal growth [16].

Prototypes providing MICs under ≤32 µg/mL against *S. brasiliensis* were considered to be promising candidates for future antifungal drug development. Three independent experiments were performed for all assays.

### 4.5. In Silico Toxicity and Pharmacological Profiles

In silico pharmacokinetic properties and toxicity estimations (ADMET) were evaluated using pkCSM—pharmacokinetic web server (http://biosig.unimelb.edu.au/pkcsm/ (accessed on 21 June 2021) [49], OSIRIS Property Explorer [56] and SwissADME webserver [57]. These results were compared with the itraconazole profile. The evaluated theoretical pharmacokinetic properties were absorption, distribution, metabolism and excretion. Absorption suggests a theoretical human intestinal absorption due to the Caco-2 permeability, P-glycoprotein substrate and P-glycoprotein I/II inhibition. For distribution, the theoretical steady-state volume of distribution was analyzed, the blood–brain barrier penetration (BBBP) and the fraction unbound to serum proteins in humans. The metabolism analyses were based on the relationships with cytochrome P450 (CYP) enzymes: the inhibition of CYP1A2, CYP3A4, CYP2C19, CYP2C9 and CYP2D6, and as the substrate of CYP3A4 and CYP2D6. Lastly, excretion parameters were assessed by the total theoretical clearance and renal OCT2 substrate. Toxicological analyses comprise toxicity target, hepatotoxicity, hERGI/II inhibitors and the toxicity end points such as carcinogenic, mutagenic, immunotoxic and cytotoxic parameters.

## 5. Conclusions

This original work was based on the premise of finding new potent and promising compounds with antifungal activity, based on the fact that antifungal armamentarium is very limited and many fungal species are resistant to clinically available drugs. Thus, our original aim was to evaluate the possible antifungal action of novel compounds, including those from the hydrazone and quinone classes, which are well-recognized molecules with several biological properties such as antimicrobial potential. Three hydrazone derivatives (H1, H2 and H3) showed good in vitro and in silico performances against the yeast pathogenic phase of the dimorphic fungi *S. brasiliensis*, compared with those of other promising antifungal drug candidates. These findings are particularly relevant if the results against itraconazole-resistant isolates, originally from non-responsive cats, are to be considered. Furthermore, although understanding the mechanisms of action and the antifungal drug targets are important, future studies of the mechanisms involved in *S. brasiliensis* drug resistance are necessary. Our results set the grounds for the discovery of novel promising compounds which can be used as a platform for the synthesis of more potent derivatives. Finally, since sporotrichosis is a neglected zoonosis currently spreading in Brazil and Latin America, the present data can contribute to its future control by alternative antifungal drug design against *S. brasiliensis*—the most virulent and prevalent species of the hyperendemic scenario.

## Figures and Tables

**Table 1:** Clinical epidemiological and laboratorial data of domestic cats infected by the six *S. brasiliensis* isolates investigated in the present study.

Clinical/Epidemiological	Wild Type			Non-Wild Type		
	WT1	WT2	WT3	NWT1	NWT2	NWT3
Age (years)	10	5	2.5	2	1	1
Sex	M	F	M	M	M	F
Castration	Yes	No	No	Yes	No	Yes
Free roaming	No	Yes	Yes	Yes	Yes	Yes
Anatomical site	Abdomen, head, chest and pelvic limb	Head and neck	Head, thoracic and pelvic limb	Base of tail and paw	Head	Pelvic limb
Relapse	No	No	Yes	Yes	No	No
Duration of ITC treatment (months)	60	NI	8	>120	NI	2

WT: wild type; NWT: non-wild type to ITC [23]; M: male; F: female; ITC: itraconazole; NI: not informed.

**Table 2:** In vitro susceptibility (µg/mL) of the six isolated *Sporothrix brasiliensis* (yeast forms) to the novel hydrazone derivatives.

Azole/Hydrazones		ATCC/Clinical Isolates						
		*S. bra*	WT 1	WT 2	WT 3	NWT 1	NWT 2	NWT 3
ITC	MIC	2	1	4	1	32	16	8
	MFC	16	8	32	8	>128	128	128
H1	MIC	8	2	4	2	1	8	8
	MFC	4	2	8	2	2	16	8
H2	MIC	8	16	16	8	8	16	8
	MFC	8	16	16	8	16	16	16
H3	MIC	4	2	4	16	1	16	1
	MFC	4	4	8	16	1	16	1

*Sbra*: *Sporothrix brasiliensis* (ATCC MYA-4823); MIC: minimum inhibitory concentration; MFC: minimum fungicidal concentration.

**Table 3:** Geometric means generated from the in vitro susceptibility (µg/mL) of the six isolated *Sporothrix brasiliensis* (yeast forms) to novel hydrazone derivatives.

Azole/Hydrazones		ATCC/Clinical Isolates		
		*S. bra*	WT GM	NWT GM
ITC	MIC	2	2	18.6
	MFC	16	16	128
H1	MIC	8	2.7	5.7
	MFC	4	4	8.7
H2	MIC	8	13.3	10.7
	MFC	8	13.3	16
H3	MIC	4	7.3	6
	MFC	4	9.3	6

*Sbra*: *Sporothrix brasiliensis* (ATCC MYA-4823); MIC: minimum inhibitory concentration; MFC: minimum fungicidal concentration; GM: geometric mean.

**Table 4:** In vitro susceptibility (µg/mL) of the six isolated *Sporothrix brasiliensis* (yeast form) to novel quinone derivatives.

Azole/Quinones		ATCC/Clinical Isolates						
		*S. bra*	WT 1	WT 2	WT 3	NWT 1	NWT 2	NWT 3
Itraconazole	MIC	2	1	4	1	32	16	8
	MFC	16	8	32	8	>128	128	128
Q1	MIC	>128	32	>128	NA	128	NA	64
	MFC	>128	32	>128	NA	128	NA	64
Q2	MIC	>128	64	NA	NA	>128	NA	64
	MFC	>128	64	NA	NA	>128	NA	128
Q3	MIC	>128	64	NA	NA	>128	NA	128
	MFC	>128	128	NA	NA	>128	NA	128
Q4	MIC	>128	128	NA	NA	>128	>128	128
	MFC	>128	128	NA	NA	>128	>128	128
Q5	MIC	>128	NA	NA	NA	NA	NA	128
	MFC	>128	NA	NA	NA	NA	NA	128
Q6	MIC	NA	128	NA	NA	NA	NA	64
	MFC	NA	128	NA	NA	NA	NA	64
Q7	MIC	NA	128	NA	NA	NA	NA	64
	MFC	NA	128	NA	NA	NA	NA	64
Q8	MIC	32	128	>128	NA	32	>128	64
	MFC	64	128	>128	NA	32	>128	64
Q9	MIC	>128	128	NA	NA	NA	NA	128
	MFC	>128	128	NA	NA	NA	NA	128
Q10	MIC	128	128	NA	NA	NA	NA	128
	MFC	128	128	NA	NA	NA	NA	128
Q11	MIC	128	64	NA	NA	NA	NA	128
	MFC	128	64	NA	NA	NA	NA	128

*Sbra*: *Sporothrix brasiliensis* (ATCC MYA-4823); MIC: minimum inhibitory concentration; MFC: minimum fungicidal concentration; NA: not analyzed due to the lack of evidence after TSA screening.

**Table 5:** Toxicological in silico profile of synthetic derivatives.

Compounds	Toxicity									
	Oral Rat Acute Toxicity (LD50)	Oral Rat Chronic Toxicity (LOAEL)	Minnow Toxicity	HERG I	HERG II	Hepatotoxicity	Toxicological End Points			
							Immunotoxicity	Carcinogenicity	Cytotoxicity	Mutagenicity
	Numeric (mol/kg)	Numeric (log mg/kg_bw/day)	Numeric (log LC 50)	Categorical (Yes/No)			Categorical (Active/Inactive)			
Itraconazole	2.966	0.055	−4.446	No	Yes	Yes	Yes	No	No	No
Q1	1.952	2.322	−2.223	No	Yes	Yes	No	No	No	No
Q2	2.098	2.398	−2.889	No	Yes	Yes	No	No	No	No
Q3	2.245	2.381	−2.098	No	Yes	Yes	No	No	No	No
Q4	2.580	1.549	−1.846	No	Yes	Yes	Yes	No	No	Yes
Q5	2.577	1.581	−1.211	No	Yes	Yes	No	No	No	Yes
Q6	2.972	1.221	0.919	No	Yes	No	No	No	No	No
Q7	2.965	2.426	0.274	No	Yes	No	No	No	No	No
Q8	2.711	2.974	−6.407	No	Yes	Yes	No	No	No	No
Q9	2.844	1.724	−0.550	No	No	Yes	No	No	No	No
Q10	2.905	1.578	−1.852	No	Yes	Yes	No	No	No	No
Q11	2.247	2.597	−2.126	No	Yes	Yes	Yes	No	No	No
H1	2.596	1.380	0.167	No	No	No	No	No	No	Yes
H2	2.549	1.340	0.644	No	No	Yes	No	No	No	No
H3	2.984	1.238	−0.396	No	No	No	No	No	No	No

Itra: itraconazole; HERG I: Type 1 human Ether-a-go-go-related gene; HERG II: Type 2 human Ether-a-go-go-related gene.

## Data Availability

All data are contained in the main text of the article.

## Supplementary Materials

The following are available online at https://www.mdpi.com/article/10.3390/ph15010055/s1, Table S1: In silico pharmacokinetics profile of the hydrazone and quinone synthetic derivatives.
