# Supplementary material for: Synthetic Derivatives against Wild-Type and Non-Wild-Type Sporothrix brasiliensis: In Vitro and In Silico Analyses
Source: Pharmaceuticals (Basel). 2022 Jan 1;15(1):55. doi: 10.3390/ph15010055 (PMC8781075; doi:10.3390/ph15010055)
Supplement: Supplementary file 1 [file pharmaceuticals-15-00055-s001.zip › pharmaceuticals-1494367-supplementary.pdf]

Table S1. *In silico* pharmacokinetics profile of the hydrazone and quinone synthetic derivatives.

Compounds	Absorption					Distribution			Metabolism							Excretion	
	Intestinal absorption (human)	Caco-2 permeability	P-glycoprotein substrate	P-glycoprotein I inhibition	P-glycoprotein II inhibition	VDss (human)	Fraction unbounded	BBB permeability	Substrate		Inhibition					Total clearance	Renal OCT2 substrate
									CYP								
	Numeric (%Absorbed)	Categorical (Yes/No)	Categorical (Yes/No)			Numeric (Log L/kg)	Numeric (Fu)	Categorical (Yes/No)	2D6	3A4	1A2	2C19	2C9	2D6	3A4	Numeric (Log ml/min/kg)	Categorical (Yes/No)
									Categorical (Yes/No)								
Itra	100	Y	Y	Y	Y	-0.167	0.239	N	N	Y	N	Y	Y	N	Y	0.056	N
Q1	96.778	Y	N	Y	Y	-0.614	0	N	N	Y	N	Y	Y	N	Y	0.058	N
Q2	97.050	Y	Y	Y	Y	-0.515	0.04	N	N	Y	N	Y	Y	N	N	0.072	N
Q3	97.220	Y	Y	Y	Y	-0.595	0.053	N	N	Y	N	Y	Y	N	Y	0.057	N
Q4	88.680	N	Y	Y	Y	-0.807	0.184	N	N	Y	N	N	Y	N	Y	0.037	N
Q5	88.515	N	Y	Y	Y	-0.791	0.174	N	N	Y	N	N	Y	N	Y	0.098	N
Q6	97.451	Y	Y	Y	Y	-0.252	0.194	N	N	Y	N	N	Y	N	Y	0.039	N
Q7	100	Y	N	Y	Y	-0.312	0.153	N	N	Y	N	N	Y	N	Y	0.035	N
Q8	100	Y	Y	Y	Y	-0.347	0.274	N	N	Y	N	N	N	N	N	-0.926	N
Q9	84.812	Y	Y	Y	N	-0.187	0.029	N	N	Y	N	N	N	N	N	0.072	N
Q10	100	Y	Y	Y	Y	-0.574	0.196	N	N	Y	N	Y	Y	N	Y	-0.045	N
Q11	99.261	N	Y	Y	Y	-0.557	0.079	N	N	Y	N	N	Y	N	Y	-0.002	N
H1	99.261	N	Y	N	N	-0.317	0.286	N	N	N	N	N	N	N	N	0.786	N
H2	93.340	N	N	N	N	-0.231	0.386	N	N	N	N	N	N	N	N	0.726	N
H3	91.647	N	N	N	N	-0.169	0.324	Y	N	N	Y	N	N	N	N	0.285	N

Itra: itraconazole; VDss: volume of distribution; FU: fraction unbounded; BBB: blood brain barrier permeability; Y: yes; N: no; HERG I: Type 1 human ether-a-go-go-related gene; HERG II: Type 1 human ether-a-go-go-related gene.
